# Supplementary material for: Somatodendritic consistency check for temporal feature segmentation
Source: Nat Commun. 2020 Mar 25;11:1554. doi: 10.1038/s41467-020-15367-w (PMC7096495; doi:10.1038/s41467-020-15367-w)
Supplement: Supplementary file 1 — Supplementary Information [file 41467_2020_15367_MOESM1_ESM.pdf]

Supplementary information:

Somatodendritic consistency check for temporal feature segmentation

Asabuki et al.

Supplementary Methods

Simulation details

In Figs 1, 2 and 3, synaptic input to an output neuron during the presentation of a target input pattern was the superposition of a target-specific Poisson spike train of rate $r_{\mathrm{sig}} = r/(1 + (S/N)^{-1})$ and a background Poisson spike train of rate $r - r_{\mathrm{sig}}$, where S/N refers to the signal-to-noise ratio $r_{\mathrm{sig}}/(r - r_{\mathrm{sig}})$. Note that $r_{\mathrm{sig}} < r$. The temporal patterns of target-specific spike trains were kept unchanged across the repetition of target patterns, whereas background input changed their temporal patterns for each repeat. Outside the target patterns, Poisson spike trains of rate r were given as input. Thus, the spike rate of synaptic input was always r, which was fixed at 5 Hz. In Fig. 2G, we did not model the realistic process of failure in synaptic transmissions, i.e., failure in evoking postsynaptic potentials by presynaptic spikes. Instead, we simulated transmission failure in the following way. We first generated Poisson spike trains with a fixed Poisson rate $r/(1 - p_{\mathrm{fail}})$, where p_{fail} is the failure probability of presynaptic transmissions. Then, assuming that the failure rate is small, we eliminated spikes with the probability p_{fail}. In Fig. 2H, we introduced trial-by-trial jitters in presynaptic spikes. First, we generated the reference spike trains used for the presentation of a chunk to individual input neurons. Then, in each presentation of the chunk, spike times were sifted by the amounts drawn by a Gaussian distribution with mean zero.

In Fig. 4, 5, the numbers of input and output neurons were 1000 and 10, respectively and each input neuron selectively responded to a letter at the firing rate of 10 Hz. In Fig. 4A and 4E, the individual chunks consisting of four English letters had the same length of 30 ms and appeared in the input sequence with the same occurrence probabilities of 1/3. Letters in each chunk appeared in a fixed order. Both feedforward connections and lateral inhibitory connections were trained.

In Fig. 6, the size of each image was 28×28 (= 784) pixels and each bar has a width of 7 pixels. Each pixel took its value on either 1 or 0. Noise was generated by flipping pixels with the probability of 0.1, and the images were blurred by circular masks to prevent artifacts from the edges of the images. Each input neuron received input from an image pixel without overlaps between neurons, and the neurons responding to the pixels of value 1 generated Poisson spike trains with the mean firing rate of 40 Hz. The total time of training was 400 sec. The weights of lateral inhibitory connections were not modifiable.

In Fig. 7 and 8, the network had 500 input neurons and two output neurons with non-modifiable lateral inhibition. Each input neuron generated a Poisson spike train at an instantaneous firing rate equal to the waveform of the mixture signal assigned randomly to the neuron. During training, the network model was repeatedly exposed to mixture signals 60 times, where each presentation had 500,000 time steps. In sampling spiking activities, we normalized the mixture signals between the minimum (0 Hz) and maximum (10 Hz) rates. Note that, input spike trains varied from trial to trial although their rate profiles were unchanged. The sampling rate of audio files was 44.1 kHz and the unit time step of network simulations was 1 ms. For comparison with FastICA, we used the FastICA function of Python library scikit-learn with a log cosh function, and tolerance on update at each iteration was 0.0001 (22, 45). FastICA is efficient and popularly used. Independence of auditory signals was evaluated with the negentropy (negative entropy), which measures the deviations of sampled signals from a Gaussian random process. For comparison with temporal ICA, we

used the Second Order Blind Identification (SOBI) algorithm, which is based on the second-order statistics of observed signals and minimizes the correlations among the time-shifted versions of given mixed signals with various delays. We used the open source software of SOBI, which is available at https://github.com/davidrigie/sobi. The learning rate of SOBI was set equal to 10^{-6}. We compared our model with FastICA and SOBI in two cases. In the first case, we used the sounds of two music instruments playing different pieces of music. In the second case, we used the sounds of the same two instruments playing their own repertoires of the same piece of music. The summed negentropy of two source signals was 0.001 and 0.0004 for the former and latter cases, respectively, indicating that the two signals were less independent in the latter case than in the former case. Generally, the two source signals were highly correlated. Our model gives output activities that are generally delayed behind the input (Fig. 7C). The delay was adjusted at about 13 ms to obtain the maximal correlations between true sources and output neuron activities. We paired each output activity with the true source that yields the largest correlation. In FastICA and SOBI, the signs of the estimated signals can be opposite to those of the true sources. Therefore, we calculated the correlations between all pairs and all possible combinations of signs (when the number of sources is two, $2! \times 2^2 = 8$ patterns) to adopt the maximal value. In Fig. 7C, each waveform was reconstructed from the firing rate of each output neuron averaged over 20 trials with an identical set of initial weights after standardization. Namely, we subtracted the temporal average of the (trial-averaged) firing rate from the instantaneous values and divided the resultant differences by the standard deviation. In Fig. 7D, simulations were performed 400 times for all combinations of 20 different sets of input spike trains and 20 different sets of initial weights.

Values of parameters used in the simulations

The values of parameters used in the present simulations are as follows: in Fig. 1, $N_{\mathrm{in}} = 2{,}000$, $N_{\mathrm{out}} = 1$, $\theta_0 = 1.7$, $\phi_0 = 10$ Hz, $\eta = 1.0 \times 10^{-6}$, $G_{\max} = 0.0$, $C_{\mathrm{p}} = C_{\mathrm{d}} = 0.0$, $\gamma = 5$ and $t_0 = 15$ sec; in Fig. 2 Supplementary Figure 1 and Supplementary Figure 2, $N_{\mathrm{in}} = 500$, $N_{\mathrm{out}} = 10$, $\theta_0 = 0.5$, $\phi_0 = 30$ Hz, $\eta = 1.0 \times 10^{-6}$, $G_{\max} = 0.1$ (2.0 in Supplementary Figure 1A and 0.01 in Supplementary Figure 1B), $C_{\mathrm{p}} = 0.00525$, $C_{\mathrm{d}} = 0.0105$, $\gamma = 5$ (0 in Supplementary Figure 1C, top and 20 in Supplementary Figure 1C, bottom) and $t_0 = 15$ sec; in Fig. 3, $N_{\mathrm{out}} = 1$, $\theta_0 = 0.5$, $\phi_0 = 10$ Hz, $\eta = 5.0 \times 10^{-7}$ $\gamma = 5$ and $t_0 = 15$ sec; in Figs. 4, 5 and Supplementary Figure 4, $N_{\mathrm{in}} = 1000$, $N_{\mathrm{out}} = 10$, $\theta_0 = 0.5$, $\phi_0 = 10$ Hz, $\eta = 1.0 \times 10^{-4}$, $G_{\max} = 0.1$, $C_{\mathrm{p}} = 0.0525$, $C_{\mathrm{d}} = 0.105$, $\gamma = 0.05$ and $t_0 = 9$ sec; in Fig. 6, $N_{\mathrm{in}} = 784$, $N_{\mathrm{out}} = 50$, $\theta_0 = 1$, $\phi_0 = 10$ Hz, $\eta = 5.0 \times 10^{-4}$, $G_{\max} = 0.07$, $C_{\mathrm{p}} = C_{\mathrm{d}} = 0.0$, $\gamma = 5$ and $t_0 = 4$ sec; in Fig. 7, 8 and Supplementary Figure 5, $N_{\mathrm{in}} = 500$, $N_{\mathrm{out}} = 2$, $\theta_0 = 0.5$, $\phi_0 = 10$ Hz, $\eta = 1.0 \times 10^{-5}$, $G_{\max} = 0.1$, $C_{\mathrm{p}} = 0.0$, $C_{\mathrm{d}} = 0.0$, $\gamma = 5$ and $t_0 = 1.5$ sec. The network was typically trained for the duration of 500 seconds.

Quantification and statistical analysis

In Fig. 2F-H, the mean and standard deviation of correlations were calculated between the responses of output neurons and chunk-specific reference responses over 20 networks with different initial conditions at each noise level. In Fig. 7D, output responses were first averaged over 20 different sets of spike trains and the correlations between the averaged responses and the original signals were calculated over 20 random initial weights.

Supplememtary Figures

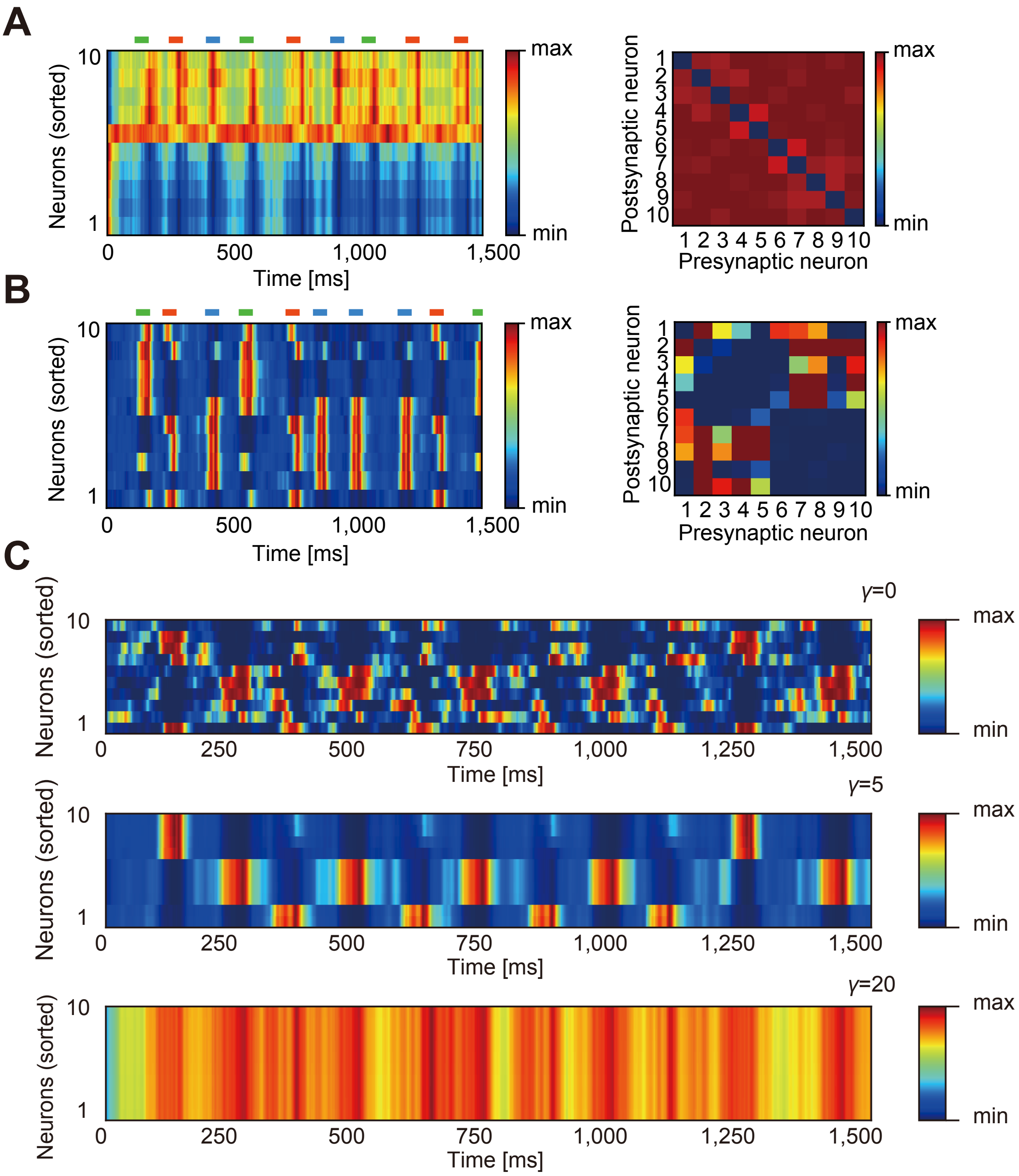

Supplementary Figure 1. Roles of inhibitory STDP and regularization parameter. Here, simulations were performed with irregular spike trains of 200 input neurons. The other settings were the same as in Figure 2. (A) Post-learning responses of output neurons are shown for strong lateral inhibition (left). About half of the neurons responded to all three chunks without stimulus selectivity, but the others showed almost no responses. Synaptic weight matrix developed no clustering structures (right). (B) Post-learning responses are shown for weak lateral inhibition (left). The majority of the neurons had more than one preferred stimulus. Synaptic weight matrix developed overlapping clustering structures (right). (C) The strength of the regularization term was changed in Equation (19) in the task shown in Figure 2. Too weak regularization term impaired the self-organization of selective responses to chunks (top), a moderate range of the regularization term resulted in successful learning (middle), and too strong regularization term prohibited the learning of features (bottom).

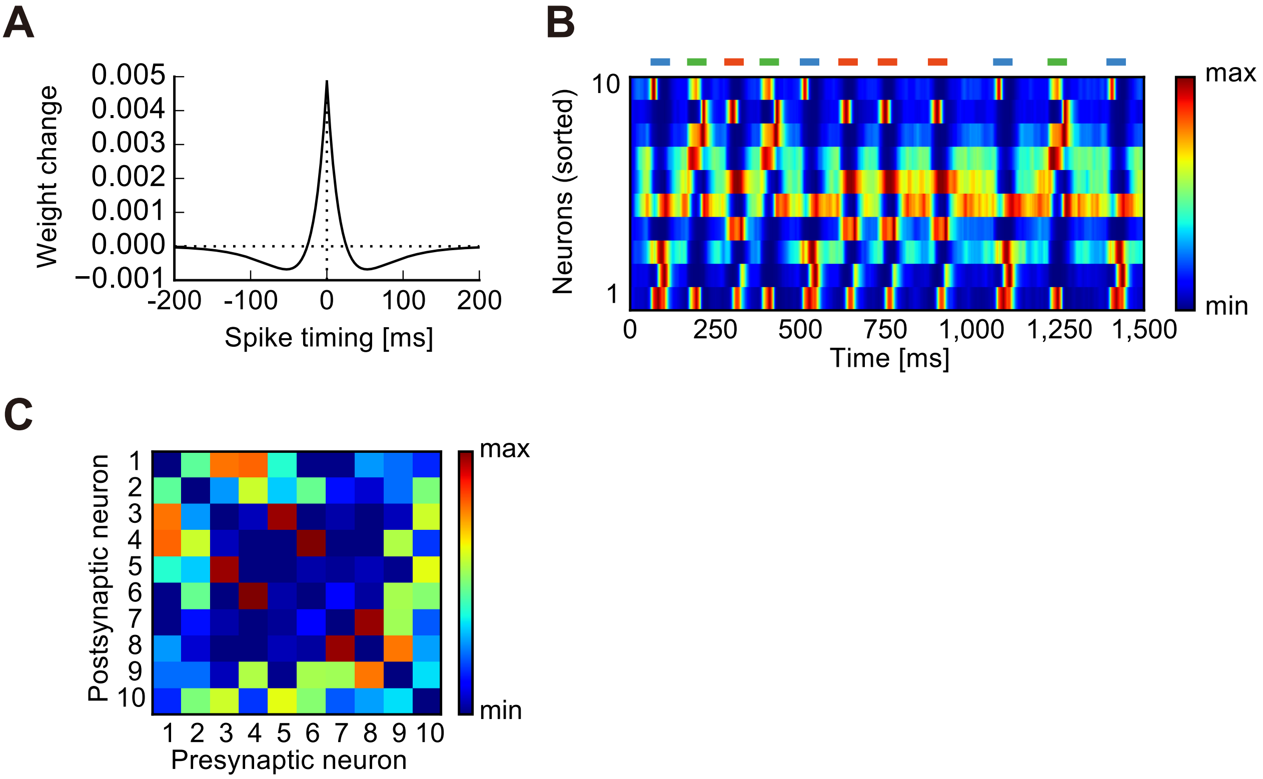

Supplementary Figure 2. Temporal feature detection by a different iSTDP rule. Input spike trains were the same as those used in Fig. 2. (A) Window function of iSTDP used in the simulations strengthens inhibition between synchronously firing neuron pairs. (B) Post-learning phasic responses of output neurons are shown. Horizontal bars show the intervals in which three chunks (green, red, and blue) were presented. (C) Post-learning synaptic weight matrix of lateral inhibition did not have an obvious cell-assembly structure.

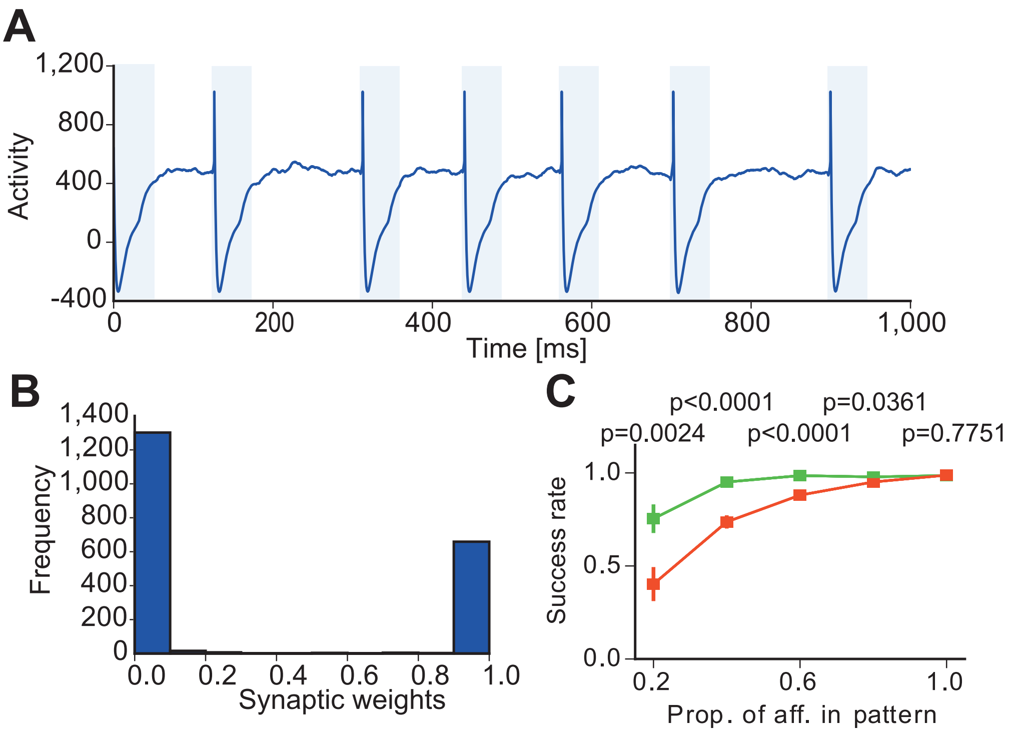

Supplementary Figure 3. Comparison with the STDP-based model in spike sequence detection. (A) A computer code was generated for the previous model according to (31). The previous model generates a single spike when it successfully detects recurring input spike patterns (shaded areas). (B) Bimodal distributions of self-organized synaptic weights. (C) Success rates are plotted for the STDP model (red) and our model (green) against the proportions of input neurons encoding the recurring pattern. Vertical bars are standard deviations and P values were calculated between the results of the two models (two-sided Welch's t-test). Each comparison was performed over 20 independent samples.

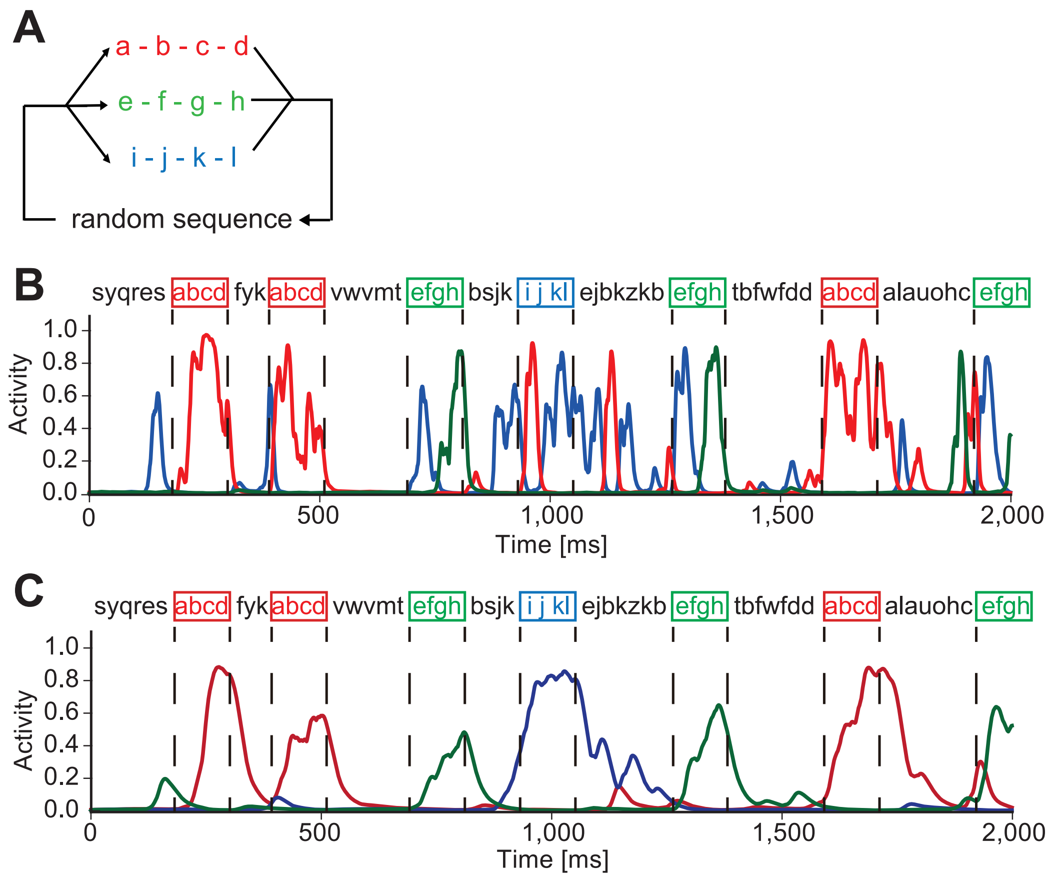

Supplementary Figure 4. Chunking in the presence of distractors. (A) Three chunks were separated by random sequences of arbitrary English characters. The length of random sequences ranged from 3 to 7. (B) Typical responses of output neurons with fast synaptic time constant (5 ms) are shown. (C) Same as (B) except that the synaptic time constant is slow (50 ms).

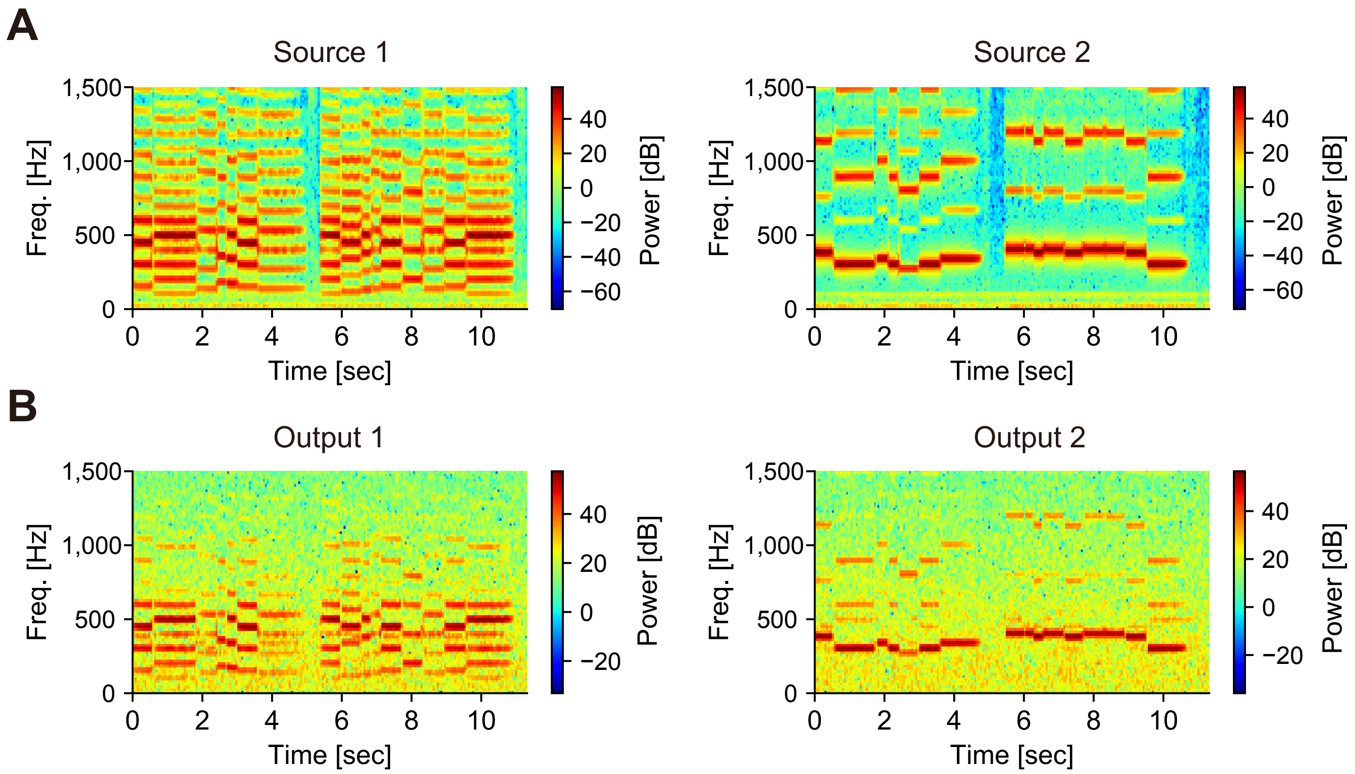

Supplementary Figure 5. Spectrogram of true and estimated signals in BSS.(A) The spectrograms of the true sources. The two sources were taken from the same piece of music. (B) Example spectrograms of the estimated signals. The network model cut off the high-frequency components above ~ 60 Hz. This was because the membrane dynamics act as a low-pass filter with the cut-off frequency being the inverse of the membrane time constant.

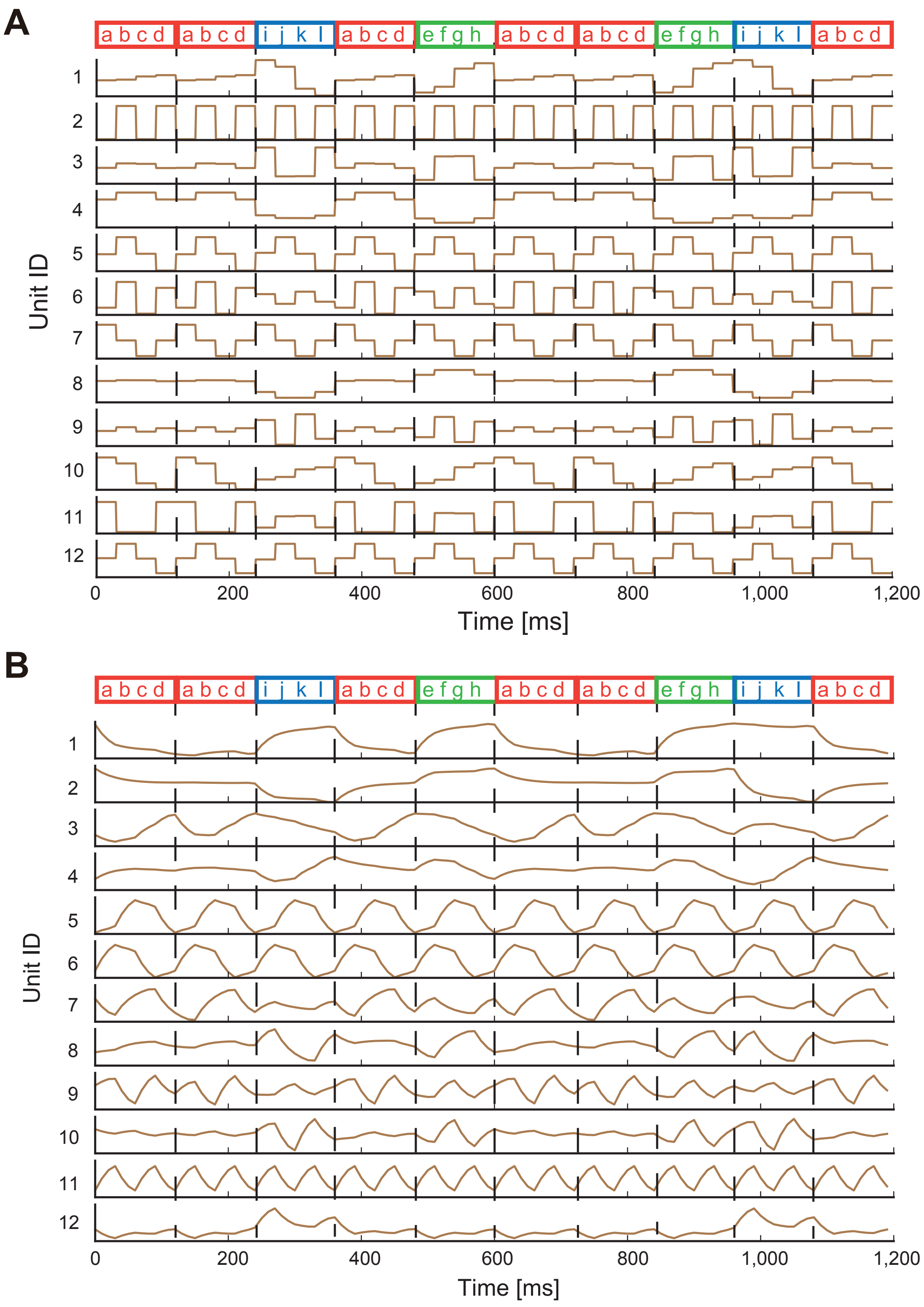

Supplementary Figure 6. Chunking of character sequences by SOBI. We attempted to learn three chunks each consisting four non-overlapping English characters. (A) Post-learning outputs of 12 output units are shown. Note that the number of output units needs to be coincide with that of English characters involved in inputs (a, b, … , l) because SOBI is derived based on a linear algebra. (B) This example shows output responses for a low-pass filtered version of the input used in A. The input was low-pass filtered by slow synaptic current with the decay constant of 50 ms and then binned by 10 ms. Examples for different parameter values are not shown as the essential results were unchanged.
