# Supplementary material for: Somatodendritic consistency check for temporal feature segmentation
Source: Nat Commun. 2020 Mar 25;11:1554. doi: 10.1038/s41467-020-15367-w (PMC7096495; doi:10.1038/s41467-020-15367-w)
Supplement: Supplementary file 3 — Reporting Summary [file 41467_2020_15367_MOESM3_ESM.pdf]

nature research

Corresponding author(s): Tomoki Fukai

Last updated by author(s): Nov 11, 2019

Reporting Summary

Nature Research wishes to improve the reproducibility of the work that we publish. This form provides structure for consistency and transparency in reporting. For further information on Nature Research policies, see Authors & Referees and the Editorial Policy Checklist .

Statistics

For all statistical analyses, confirm that the following items are present in the figure legend, table legend, main text, or Methods section.

n/a	Confirmed	
☐	☒	The exact sample size (*n*) for each experimental group/condition, given as a discrete number and unit of measurement
☐	☒	A statement on whether measurements were taken from distinct samples or whether the same sample was measured repeatedly
☐	☒	The statistical test(s) used AND whether they are one- or two-sided<br>*Only common tests should be described solely by name; describe more complex techniques in the Methods section.*
☒	☐	A description of all covariates tested
☒	☐	A description of any assumptions or corrections, such as tests of normality and adjustment for multiple comparisons
☐	☒	A full description of the statistical parameters including central tendency (e.g. means) or other basic estimates (e.g. regression coefficient) AND variation (e.g. standard deviation) or associated estimates of uncertainty (e.g. confidence intervals)
☐	☒	For null hypothesis testing, the test statistic (e.g. *F*, *t*, *r*) with confidence intervals, effect sizes, degrees of freedom and *P* value noted<br>*Give P values as exact values whenever suitable.*
☒	☐	For Bayesian analysis, information on the choice of priors and Markov chain Monte Carlo settings
☒	☐	For hierarchical and complex designs, identification of the appropriate level for tests and full reporting of outcomes
☐	☒	Estimates of effect sizes (e.g. Cohen's *d*, Pearson's *r*), indicating how they were calculated

Our web collection on statistics for biologists contains articles on many of the points above.

Software and code

Policy information about availability of computer code

Data collection	python3, numpy 1.17.3, scipy 0.18.1, Modular toolkit for Data Processing, open source software of SOBI (available at https://github.com/davidrigie/sobi). Our implementation is now open to public and available at https://github.com/ToshitakeAsabuki/MRIL_codes.
Data analysis	python3, numpy 1.17.3, scipy 0.18.1,

For manuscripts utilizing custom algorithms or software that are central to the research but not yet described in published literature, software must be made available to editors/reviewers. We strongly encourage code deposition in a community repository (e.g. GitHub). See the Nature Research guidelines for submitting code & software for further information.

Data

Policy information about availability of data

All manuscripts must include a data availability statement. This statement should provide the following information, where applicable:

- Accession codes, unique identifiers, or web links for publicly available datasets
- A list of figures that have associated raw data
- A description of any restrictions on data availability

All numerical datasets necessary to replicate the results shown in this article can easily be generated by numerical simulations with the software code available at available at https://github.com/ToshitakeAsabuki/MRIL_codes. No datasets were generated during this study.

Field-specific reporting

Please select the one below that is the best fit for your research. If you are not sure, read the appropriate sections before making your selection.

Life sciences study design

All studies must disclose on these points even when the disclosure is negative.

Sample size	This is a theoretical study and all data were generated by computer simulation. Sample sizes were determined to be sufficiently large for obtaining robust results within a reasonable computation time (while we tested with the sample size of more than 40 in some analysis, the essential results didn't change). Thus, in many simulation results, the sample sizes were set in the range of 5-20.
Data exclusions	No exclusions.
Replication	We ran multiple simulations with different initial conditions for network configuration and neuron states to verify the robustness of results.
Randomization	Not relevant. In all of our simulations, we initialized parameters and input sequences at random, thus there must be no biases.
Blinding	Not relevant. In all of our simulations, we initialized parameters and input sequences at random, thus there must be no arbitrariness.

Reporting for specific materials, systems and methods

We require information from authors about some types of materials, experimental systems and methods used in many studies. Here, indicate whether each material, system or method listed is relevant to your study. If you are not sure if a list item applies to your research, read the appropriate section before selecting a response.

Materials & experimental systems

n/a	Involved in the study
☒	☐ Antibodies
☒	☐ Eukaryotic cell lines
☒	☐ Palaeontology
☒	☐ Animals and other organisms
☒	☐ Human research participants
☒	☐ Clinical data

Methods

n/a	Involved in the study
☒	☐ ChIP-seq
☒	☐ Flow cytometry
☒	☐ MRI-based neuroimaging
