# Supplementary material for: Somatodendritic consistency check for temporal feature segmentation
Source: Nat Commun. 2020 Mar 25;11:1554. doi: 10.1038/s41467-020-15367-w (PMC7096495; doi:10.1038/s41467-020-15367-w)
Supplement: Supplementary file 4 — Description of Additional Supplementary Files [file 41467_2020_15367_MOESM4_ESM.pdf]

Description of Additional Supplementary Files

File Name: Supplementary Audio 1
Description: An example of mixture sounds of a bassoon and a clarinet playing their repertoires of an identical piece of music, followed by the original sounds of the two instruments before mixing. The original sounds were obtained from the Bach 10 dataset generated in Duan, Z. & Pardo, B. Soundprism: an online system for score-informed source separation of music audio, *IEEE Journal of Selected Topics in Signal Process* **5**, 1205-1215 (2011).

File Name: Supplementary Audio 2
Description: The sound of bassoon separated by our model from the mixture sounds, which we generated from the original sounds provided in Duan, Z. & Pardo, B. Soundprism: an online system for score-informed source separation of music audio, *IEEE Journal of Selected Topics in Signal Process* **5**, 1205-1215 (2011).

File Name: Supplementary Audio 3
Description: The sound of clarinet separated by our model from the mixture sounds. The original sounds before mixing were provided in Duan, Z. & Pardo, B. Soundprism: an online system for score-informed source separation of music audio, *IEEE Journal of Selected Topics in Signal Process* **5**, 1205-1215 (2011).

File Name: Supplementary Audio 4
Description: One of the signals separated by FastICA from the mixture sounds. The original sounds were provided in Duan, Z. & Pardo, B. Soundprism: an online system for score-informed source separation of music audio, *IEEE Journal of Selected Topics in Signal Process* **5**, 1205-1215 (2011).

File Name: Supplementary Audio 5
Description: The sound of bassoon separated by SOBI from the mixtures of the original sounds provided in Duan, Z. & Pardo, B. Soundprism: an online system for score-informed source separation of music audio, *IEEE Journal of Selected Topics in Signal Process* **5**, 1205-1215 (2011).

File Name: Supplementary Audio 6
Description: The sound of clarinet separated by SOBI from the mixtures of the original sounds provided in Duan, Z. & Pardo, B. Soundprism: an online system for score-informed

source separation of music audio, *IEEE Journal of Selected Topics in Signal Process* **5**, 1205-1215 (2011).
